# Supplementary material for: Analysis of Olive Oil Mill Wastewater from Conventionally Farmed Olives: Chemical and Microbiological Safety and Polyphenolic Profile for Possible Use in Food Product Functionalization
Source: Foods. 2025 Jan 30;14(3):449. doi: 10.3390/foods14030449 (PMC11817457; doi:10.3390/foods14030449)
Supplement: Supplementary file 1 [file foods-14-00449-s001.zip › foods-3396648-supplementary.pdf]

foods MDPI

Supplementary Materials

Analysis of Olive Oil Mill Wastewater from Conventionally Farmed Olives: Chemical and Microbiological Safety and Polyphenolic Profile for Possible Use in Food Product Functionalization

Lino Sciurba [1], Serena Indelicato [2], Raimondo Gaglio [1], Marcella Barbera [3,*], Francesco P. Marra [1], David Bongiorno [2], Salvatore Davino [1], Daniela Piazzese [3,4,*], Luca Settanni [1], Giuseppe Avellone [2]

1 Department of Agricultural, Food and Forest Sciences, University of Palermo, Viale delle Scienze, Bldg. 5. 90128 Palermo. Italy
2 Department of Biological, Chemical and Pharmaceutical Science and Technology (STEBICEF), University of Palermo, Via Archirafi, 90123, Palermo, Italy
3 Department of Earth and Marine Sciences (DiSTeM), University of Palermo, Via Archirafi, 90123, Palermo, Italy
4 Centro Sostenibilità e Transizione Ecologica (CSTE), Piazza Marina 61, 90100 Palermo, Italy
* Correspondence: marcella.barbera@unipa.it (M. Barbera), daniela.piazzese@unipa.it (D. Piazzese).

Table S1. Mass transitions of target analytes in MRM mode.

Analytes	Precursor Ion	Product ion *First transition*	Product Ion *Second transition*
Acephate	184	143	49
Acetamiprid	223	126	56
Aldicarbs sulfone	223	86	148
Aldicarb – sulfoxide	207	132	89
Amtrina	228	186	68
Amitraz	294	148	91
Asulam	231	158	92
Atrazine	216	174	96
Atrazione-D5	221	179	101
Atrazine-Desethyl	188	146	79
Atrazine-desisopropyl	174	96	132
Azinphos-Ethyl	368	132	77
Azinphos-Methyl	340	132	160
Azoxystrobin	404	329	372
Benalaxyl	326	148	90
Benfuracarb	411	195	252
Bifenazate	301	198	170
Boscalid	343	307	140
Bromacil	261	205	188
Bupirimate	317	166	108

Buprofezin	306	201	57
Cadusafos	271	159	131
Carbaryl	202	145	117
Carbendazim	192	132	160
Carbofuran	222	123	165
Chlorantraniliprole	484	453	286
Chlorfenvinphos	359	155	99
Chlorpyrifos-ethyl	350	198	97
Chlorpyrifos-methyl	322	125	290
Chlortoluron	213	72	46
Cibutrina	254	198	108
Clodinafop-propargyl	350	266	91
Clomazone	240	125	89
Clorsulfuron	358	141	167
Clothianidin	250	169	132
Coumaphos	363	209	127
Cyantraniprole	475	286	-
Cyazofamid	325	108	261
Cycloxidim	326	280	180
Cymoxanil	199	128	111
Cyproconazol	292	70	125
Cyprodinil	226	108	93
Cyromazine	167	60	108
Demeton-S-mettl-sulfon	263	169	121
Diazinon	305	169	97
Dichlorvos	221	109	79
Diphenamid	240	134	167
Difenoconazolo	406	251	111
Diflufenican	395	266	246
Dimethoate	230	199	125
Diuron	233	72	46
Ethofumesate	287	259	121
Etofenprox	394	107	177
Etoxazole	360	141	57
Famoxadone	392	331	238
Fenamidone	312	236	92
Fenamiphos	304	217	202
Fenarimol	331	268	81
Fenbuconazole	337	70	125
Fenhexamid	302	97	55
Fenpyrazamide	332	230	105
Fenpyroximat	422	366	138

Fenthion	279	169	247
Flonicamid	230	202	148
Florasulam	360	129	109
Fluazifop-P-Butyl	384	282	328
Flufenacet	364	152	194
Flufenoxuron	489	158	141
Fluopicolide	383	173	109
Fluopyram	397	173	145
Fluroxypyr	255	181	209
Forchlorfenuron	248	129	93
Fosthiazate	284	228	104
Furalaxyl	302	242	95
Heptenophos	251	127	109
Hexythiazox	353	228	168
Imazalil	297	159	69
Imazamox	306	261	193
Imazapyr	262	69	149
Indoxacarb	528	150	203
Imidacloprid	256	209	175
Iprovalicarb	321	119	203
Isoproturon	207	72	46
Isoxaflutole	360	251	220
Lenacil	235	152	136
Linuron	249	182	160
Lufenuron	511	158	141
Mandipropamid	412	328	125
Mepanipyrim	224	106	77
Mesosulfuron-Methyl	504	182	83
Metalaxyl	280	220	192
Metamidofos	142	125	94
Metamitron	203	175	104
Metazachlor	278	134	210
Metconazolo	320	70	125
Methabenzthiazuron	222	149	165
Methiocarb	226	169	121
Methomyl	163	88	106
Metolachlor	284	252	176
Metoxuron	229	72	156
Metrafenone	409	209	227
Metribuzin	215	89	131
Metsulfuron-Methyl	382	167	199
Mevinphos	225	127	193

Monocrotophos	225	127	99
Monolinuron	215	126	99
Myclobutanil	289	70	125
Napropamide	272	129	171
Nicosulfuron	411	182	106
Omethoate	214	183	125
Oxadixyl	279	219	132
Oxamyl	237	72	90
Oxydemeton-methyl	247	169	109
Paclobutrazol	294	70	125
Parathion-methyl	263	108.9	79
Penconazol	284	70	159
Pencycuron	329	125	218
Pendimethalin	282	212	194
Penthiopirad	360	276	177
Phosmet	318	160	133
Picoxystrobin	368	145	205
Pinoxaden	401	317	131
Pirimicarb	239	182	72
Pirimiphos-Methyl	306	164	108
Peocloraz	376	308	266
Prometryn	242	158	200
Propamocarb	189	102	144
Propanil	218	162	127
Propiconazole	342	159	69
Propoxur	210	168	111
Propoxycarbazone sodium	421	180	138
Propyzamide	256	190	173
Pymetrozin	218	105	79
Pyraclostrobin	388	194	163
Pyrazophos	374	222	194
Pyrimethanil	200	107	82
Pyriproxifen	322	96	227
Quinoxyfen	308	198	162
Qizalofop-ethyl	373	299	91
Rimsulfuron	432	325	182
Rotenone	395	192	213
Simazine	202	132	124
Spirodiclofen	411	313	71
Spirotetramat	374	302	330
Spiroxamine	298	144	100
Tebuconazole	308	70	125

Tebufenpyrad	334	117	145
Terbuthylazine	230	174	132
Terbuthylazin-Desethyl	202	146	79
Terbutryn	242	186	91
Tetraconazole	372	159	70
Thiabendazole	202	175	131
Thiacloprid	253	90	125
Thiamethoxam	292	211	132
Thiencarbazone-methyl	391	130	359
Thifensulfuron-methyl	388	167	56
Thiodicarb	355	88	108
Thiophanate-methyl	343	151	93
Triadimefon	294	197	70
Triasulfuron	402	167	141
Triazamate	315	72	226
Triazophos	314	162	119
Tribenuron-methyl	396	155	181
Trichlorfon	257	109	221
Trifloxystrobin	409	186	145
Zoxamide	336	187	160

Table S2. LC-MS/MS parameters for phenolic compounds identified in olive oil mill wastewater samples.

	Precursor Ion (m/z) [M-H]	Product Ion (m/z)	Collision Energy (V)	RF Lens (V)
Gallic Acid	169	79	24	101
	169	125	14	101
Vanillic Acid	177	123	20	105
	177	152	20	105
Ferulic Acid	193	134	15	99
	193	178	13	99
Chlorogenic Acid	353	179	45	180
	353	191	45	180
Catechin	289	203	20	147
	289	245	15	147
Mandelic Acid	151	77	18	65
	151	107	10	65
Gentisic Acid	153	108	22	90
	153	109	14	90
Syringic Acid	197	153	12	100
	197	182	14	100
Caffeic Acid	179	107	25	101
	179	135	16	103
Trans-OH-Cynnamic	163	93	31	90
	163	119	14	90
Rutin	609	271	60	299
	609	300	38	299
Apigenin-7Glu	433	269	20	123
	433	271	20	123
Quercetin	301	151	18	166
	301	179	21	166
Kaempferol	285	202	20	195
	285	239	29	195
Hydroxytyrosol	153	95	21	97
	153	123	14	97
Cumaric Acid	163	93	31	91
	163	119	13	91
Luteolin	285	133	35	187
	285	175	26	187
Apigenin	269	117	35	178
	269	151	25	178

Oleacein	319	165	6	122
	319	195	6	122
Oleocanthal	303	165	9	92
	303	285	6	92
